# Relieving Chronic Musculoskeletal Pain in Older Adults Using Transcranial Direct Current Stimulation: Effects on Pain Intensity, Quality, and Pain-Related Outcomes

**DOI:** 10.3389/fpain.2022.817984

**Published:** 2022-04-14

**Authors:** Marie-Philippe Harvey, Marylie Martel, Francis Houde, Inès Daguet, Eléonor Riesco, Guillaume Léonard

**Affiliations:** ^1^Research Center on Aging, CIUSSS de l'Estrie-CHUS, Sherbrooke, QC, Canada; ^2^Faculté de médecine et des sciences de la santé, Université de Sherbrooke, Sherbrooke, QC, Canada; ^3^Faculté des sciences de l'activité physique, Université de Sherbrooke, Sherbrooke, QC, Canada; ^4^École de réadaptation, Université de Sherbrooke, Sherbrooke, QC, Canada

**Keywords:** transcranial direct current stimulation, brain stimulation, pain, elderly, aging

## Abstract

**Introduction:**

Chronic pain is a significant health problem and is particularly prevalent amongst the elderly. Transcranial direct current stimulation (tDCS) is a non-invasive brain stimulation technique that has been proposed to reduce chronic pain. The aim of this study was to evaluate and compare the efficacy of active and sham tDCS in reducing pain in older individuals living with chronic musculoskeletal pain.

**Materials and Methods:**

Twenty-four older individuals (mean age: 68 ± 7 years) suffering from chronic musculoskeletal pain were randomized to receive either anodal tDCS over the contralateral motor cortex (2 mA, 20 min; *n* = 12) or sham tDCS (20 min; *n* = 12) for five consecutive days. Pain logbooks were used to measure pain intensity. Questionnaires (McGill Pain Questionnaire, Brief Pain Inventory, Beck Depression Inventory [BDI], Beck Anxiety Inventory, Pain Catastrophizing Scale [PCS], and Margolis Pain Drawing and Scoring System [MPDSS]) were also used to assess pain in its globality.

**Results:**

Analysis of pain logbooks revealed that active tDCS led to a reduction in daily average pain intensity (all *p* ≤ 0.04), while sham tDCS did not produce any change (*p* = 0.15). Between-group comparisons for change in pain intensity reduction between active and sham tDCS showed a trend during treatment (*p* = 0.08) which was significant at the follow-up period (*p* = 0.02). Active tDCS also improved scores of all questionnaires (all *p* ≤ 0.02), while sham tDCS only reduced MPDSS scores (*p* = 0.04). Between-group comparisons for the pain-related outcomes showed significant differences for BDI et PCS after the last tDCS session.

**Conclusions:**

These results suggest that anodal tDCS applied over the primary motor cortex is an effective modality to decrease pain in older individuals. tDCS can also improve other key outcomes, such as physical and emotional functioning, and catastrophic thinking.

## Introduction

Chronic musculoskeletal pain is the leading cause of disability among older adults, exceeding heart diseases, strokes, respiratory conditions, and injuries (1, 2). It is, therefore, no surprise that pain, mainly musculoskeletal, is the most common reason for seeking medical care (3–5). More than a mere symptom, chronic pain is now recognized worldwide as a significant health problem having a major impact on physical functioning and quality of life (6, 7). In older adults, chronic pain is associated with decreased mobility and cognitive functioning, along with increased anxiety, depression, and loneliness (8–12). These negative effects can lead to a loss of autonomy and precipitate institutionalization (13–15).

Conservative treatments, including the use of pharmacological agents, remain one of the first line of treatment to alleviate chronic pain in late adulthood (16–19). Despite the availability of many pharmacological treatments, they are often not sufficient to relieve pain in this population (20, 21). Besides, polypharmacy remains an important problem in older individuals (19, 22–24), with numbers showing that ~20% of them suffer from undesirable drug interactions or reactions (23). In view of this, recommendations and clinical practical guidelines insist on the importance of multimodal analgesic approaches, combining both pharmacologic and nonpharmacologic treatments (13, 18, 25).

Non-invasive brain stimulation techniques, in particular transcranial direct current stimulation (tDCS), have been extensively studied in the past years as a potential nonpharmacologic approach for reducing chronic pain (26, 27). Although few investigations were conducted in the elderly [mostly patients suffering from osteoarthritis [OA] pain (28–31)], studies looking into the efficacy of tDCS for chronic pain conditions were mainly performed in middle-aged adults suffering from neuropathic pain (32–34), making it difficult to draw conclusions about the utility of such an approach in older populations. The present study aimed to fill this knowledge gap and document the effect of tDCS in older individuals living with chronic musculoskeletal pain. More specifically, we aimed to evaluate the efficacy of active tDCS in reducing pain intensity and other pain-related outcomes including pain quality, the impact of pain on physical and emotional functioning, catastrophic thinking, and body surface covered by pain.

## Materials and Methods

### Participants

**Figure 1:**
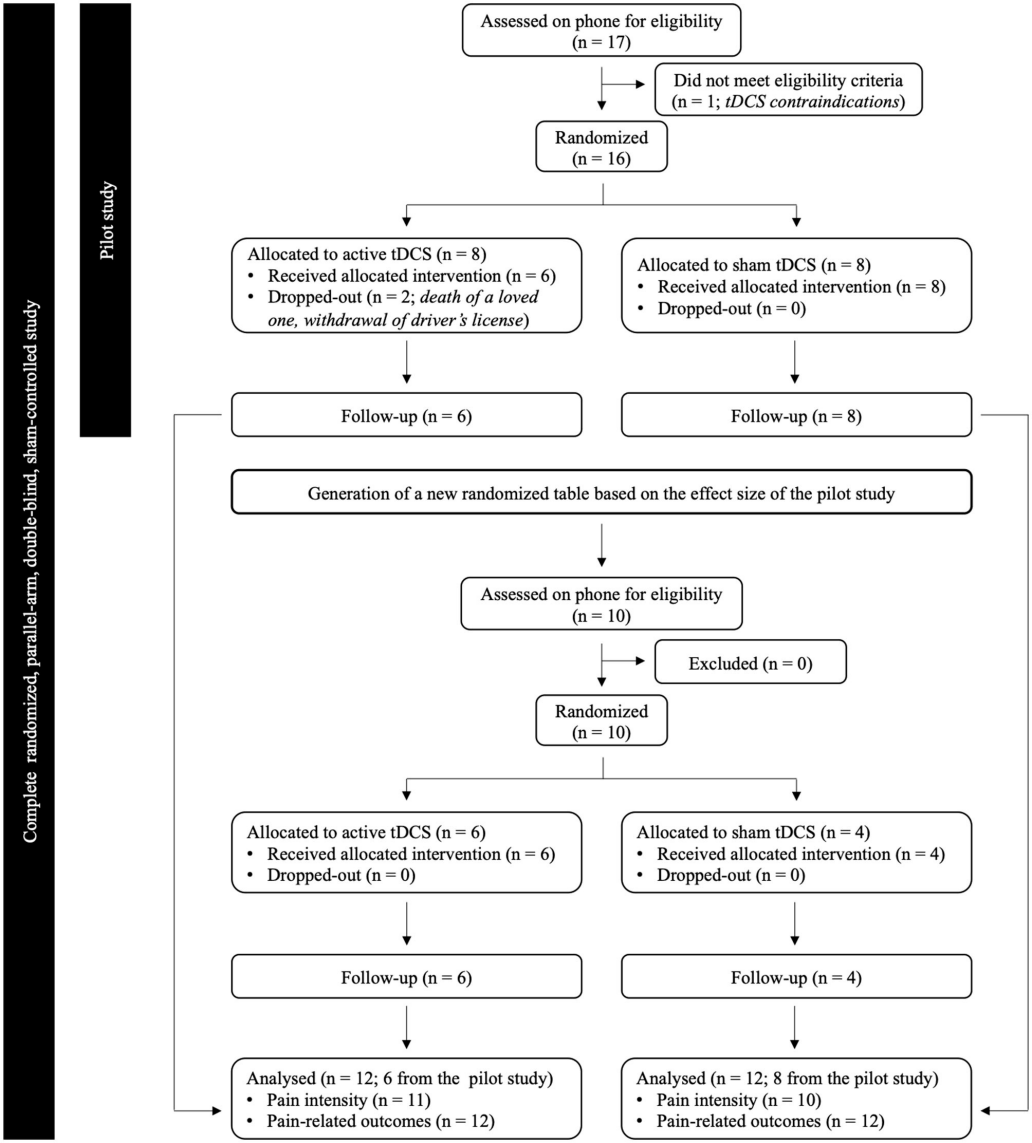
Flowchart showing recruitment and progress through the study.

Twenty-four elderly individuals were included in the study of whom 14 were part of a previously published pilot study (35). The flowchart of the study is presented in Figure 1. Individuals were regarded as suitable to participate if they fulfilled the following criteria: (1) aged 60 years or over; (2) reported stable musculoskeletal pain in the previous 3 months or more; and (3) had never undergone tDCS before. Participants with tDCS contraindications, such as psychiatric or neurological conditions (e.g., stroke, traumatic brain injury, etc.), history of brain surgery or tumor, metallic implants, epilepsy, or history of substance abuse or dependence, were excluded (36, 37). Participants reported the diagnoses they received from their primary care physician. Participants were asked to keep their medication and life habits stable for the duration of the study, and were also asked not to consume psychostimulants (nicotine and caffeine) at least six hours before testing, to avoid potential effect on pain measures (38). The experiment took place at the Research Center on Aging of the CIUSSS de l'Estrie-CHUS (Sherbrooke, Quebec, Canada). Participants were all French-speaking community-dwelling individuals. The study was approved by the local institutional ethics committee and written informed consent was obtained from all participants.

### Experimental Design

A randomized, parallel-arm, double-blind, sham-controlled design was used. The study lasted 19 days and was divided into 3 phases: a 7-day baseline evaluation (T1); a 1-week treatment period, which consisted of five consecutive daily treatment sessions of sham or active tDCS (T2); and a 7-day follow-up period (T3). Throughout the 19 days of the study, daily measures of pain were recorded with a pain logbook. Randomization to sham or active tDCS was performed using a random numbers table with a ratio of 1:1 based on the order of entry of the participants in the study created by the local institutional statistician. The randomized table design for our previous pilot study was extended to assign a total of 24 individuals (12 in each group); this new sample size was based on the calculations following the results in pain reduction obtained from the pilot study (35).

### Pain Measurements

Pain intensity (primary outcome measure) was evaluated with a daily pain logbook containing two numerical rating scales (NRSs) ranging from 0 to 10 (0 = no pain; 10 = maximal pain). These two NRSs were used to evaluate the pain felt by the participant (i) on average in the past 24 h (average pain), and (ii) at its worst during the past 24 h (maximal pain). The pain assessment logbook was filled out by the participant at the end of each day throughout the duration of the study. The NRS have been shown to be reliable and valid to measure pain intensity in elderly patients with persistent pain (39). In addition to its intensity, pain has different sensory and affective qualities that need to be measured to fully describe the experience of the patient (40). This was done using the McGill Pain Questionnaire (MPQ) (41).

Pain intensity and quality measurements are essential, but they only capture a part of the pain experience and should be supplemented by other pain-related measures (39, 42–44). Following the recommendations of others from the IMMPACT group (42, 43), we assessed the impact of pain on physical and emotional functioning with the short form of the Brief Pain Inventory (SF-BPI) (45) and the Beck Depression Inventory (BDI) (46), respectively, anxiety symptoms with the Beck Anxiety Inventory (BAI) (47), catastrophic thinking with the Pain Catatrophizing Scale (PCS), (48) and painful body surface with the Margolis Pain Drawing and Scoring System (MPDSS) (42, 49–52). These questionnaires, in addition to the MPQ, were completed three times during the study: (1) before the tDCS sessions, (2) after the last tDCS session, and (3) 1 week after the end of the intervention. The validity and reliability of all questionnaires have been previously documented (41, 45, 47–49, 53–57).

### TDCS Protocol

Participants were seated comfortably in an armchair during the 5 tDCS treatment sessions. The treatments with regards to one given participant were always given by the same investigator who was different from the evaluator. The investigator was responsible for the assignment of participants into the active or sham tDCS group, keeping the evaluator and the participants blinded. During the stimulation, the investigator hid the device from the participant with a towel. For each participant, the stimulations were given at the same time of the day to get as close as possible to a spacing of 24 h between tDCS sessions. Direct current was transferred to the participant by a saline-soaked pair of surface sponge electrodes (5 x 7 cm) and delivered by a constant, battery-driven, portable tDCS device (Model 1300-A; Soterix Medical Inc, New York, NY). Participants received either anodal or sham stimulation of the primary motor cortex (M1). The anode was placed over M1, contralateral to the most painful site (C3 or C4 according to the electroencephalogram 10/20 system), and the cathode was placed on the supraorbital area contralateral to the anode. During active tDCS, a constant anodal current of 2 mA was delivered for 20 min. This anodal tDCS procedure has been shown to increase cortical excitability and reduce various types of chronic pain (58–64). For the sham stimulation, the electrodes were placed following the same montage as the active tDCS; however, current was applied only for the first and final 30 s and turned off for the remaining time. Therefore, the individual felt the ramp up and ramp down itching sensation of the current but received no current for the rest of the stimulation period. Each participant was informed that the sensations are generally and mainly perceived at the beginning and the end of the stimulation. The tDCS device was set by the manufacturer to automatically provide this type of sham stimulation. This placebo procedure has been validated as an effective blinding method for participants, but not for investigators (65–68). For this reason, the evaluator, who was different from the investigator, did not have contact with participants after stimulation except for the last day of stimulation when pain questionnaires were to be completed. Participants were also asked not to discuss or give any information about the intervention sessions to the evaluator. Blinding of the evaluator, as well as blinding of the participants, were assessed on the last day of tDCS sessions by asking the evaluator and participants to guess to which study group participants belonged. Side effects were evaluated after each tDCS session by asking the participant to report verbally if they experienced any symptoms. If applicable, the intensity of the side effects was assessed by asking the participant to classify their symptoms as mild, moderate, or severe.

### Data Analysis

Pain intensity, as evaluated by the two NRS of the daily pain logbook (average pain and maximal pain) were averaged into 3 scores, reflecting the three phases of the study (i.e., before[T1], during[T2], and after[T3] tDCS treatments). As mentioned above, T1 represents the 7 days of baseline, T2 corresponds to the 5 days of tDCS treatments, and T3 represents the 7 days of follow-up. The mean values were used for all analyses. Percentages of hypoalgesia were also calculated to directly compare the efficacy of active and sham tDCS on pain, based on the following formula: hypoalgesia ={[pain score before treatment (T1)—pain score during or after treatment (T2 or T3)] / pain score before treatment (T1)} x 100. For pain intensity, a modified intention-to-treat analysis was used to handle missing data in the pain logbook. Specifically, participants with >10% of missing data were excluded from the analyses, whereas an average of the results for each time point was used for the participants who had <10% of missing data. There was no possibility of missing data for all the pain-related outcomes since participants completed the questionnaires in the presence of the evaluator.

The study was designed to detect a clinically important difference of 2 points on an 11-point NRS (69, 70). To detect this difference with 80% power and a 5% significance level, we determined that 24 individuals had to be enrolled in the study [based on the effect size of 0.93 observed in our pilot study (35)]. Due to the low number of participants, and since visual inspection of the histograms did not allow us to assume that the data were normally distributed, nonparametric tests were used for all the statistical analyses. Specifically, *U* Mann–Whitney tests were used to compare the two groups (between-group analyses). This allowed us to evaluate if the outcome measures were different between the active and sham tDCS groups. Friedman tests and Wilcoxon Signed-Rank tests were also used to compare if the intervention affected the outcome measures in each group (intra-group analyses). All tests were performed using SPSS (version 25 for Windows, Chicago, IL), and the differences were considered significant if *p* ≤ 0.05 was obtained.

## Results

### Participants

Twenty-four older individuals aged between 60 and 84 years (mean age 68 ± 7 years; 4 men) were included in the study. Two participants from the active tDCS group dropped out of the study during the pilot study, one because of a family event (death of a loved one–did not receive any tDCS session), and one because of a personal matter (withdrawal of driver's license–received 3 tDCS sessions before dropping out). These 2 dropouts were considered in the new randomized table generated by the statistician to include a total of 24 participants (12 by group). Data from the two participants who dropped out were excluded from all analyses. The demographic and general clinical characteristics of the 24 participants are summarized in Table 1.

**Table 1:** Clinical and demographic characteristics of the participants.

	**Active tDCS**	**Sham tDCS**
Number (n)	12	12
Gender (F/M)	10/2	10/2
Hand dominance (right/left)	12/0	12/0
**Age (years)**		
Mean ± standard deviation	69 ± 7	68 ± 8
Range	60–83	60–84
Side of the most problematic pain (right/left)	9/3	7/5
**Duration of chronic pain**		
Mean ± standard deviation	22 ± 22	12 ± 10
<2 years	2	1
Between 2 and 9 years	4	5
10 years and more	6	6
**Diagnose (n)**		
Knee osteoarthritis	7	4
Chronic low back pain	3	3
Sciatica	1	1
Chronic neck pain	1	
Sprained shoulder		1
Shoulder tendonitis		1
Polymyalgia rheumatica		1
Unspecific leg pain		1

### Blinding and Side Effects

After the end of the last tDCS session, all participants were asked to guess in which treatment group they thought they belonged to (active or sham tDCS). Of the 24 participants who completed the study, 18 had no clue and 6 had an idea about the treatment they had received. From these six participants, two assumed correctly that they received active tDCS, two assumed correctly that they had sham tDCS, and 2 (1 active and 1 sham) were incorrect. Considering all 24 participants (including the 18 individuals who were forced to decide having no clue), 13 correctly identified their assignment group. The probability of obtaining equivalent or higher success rates is 0.42 under a binomial distribution *B* (*n* = 24, *p* = 0.5). Thus, we cannot conclude from participants' responses that they did better than randomly selecting their assignment group. The performance of the evaluator was similar; for the active and the sham tDCS groups; the evaluator guessed correctly for eight out of 12 participants and for six out of 12 participants, respectively. The probability of obtaining at least 14 correct answers out of 24 is estimated at 0.27 under a binomial distribution *B* (n = 24, *p* = 0.5), indicating that the evaluator, like the participants, did not do better than chance.

All participants tolerated the tDCS treatments well without experiencing any serious adverse effects. Fourteen participants out of 24 reported minor and transient side effects during or after the tDCS session. Of the 120 tDCS sessions delivered, 24 sessions (20%) were associated with minor side effects, namely mild tingling under the electrode (79%), mild headache (8%), mild sensation of bruise under the anode (4%), and moderate heat under the electrode (8%). The majority of the side effects (55%) were reported in the first tDCS session, and more than one-third of them occurred in the sham tDCS group.

### Pain Intensity Outcomes

Pain intensity measures (primary outcome) were obtained *via* the pain logbook filled daily by the participants during the 3 phases of the study. Three participants (one in the active tDCS group and two in the sham tDCS group) were excluded because >30% of missing data. For the remaining 21 participants included in the analyses, none had missing data except for one in the sham tDCS group (5% of missing data). For this participant, one of the 7 days of the follow-up period was not completed in the pain logbook; for this participant, pain intensity was calculated by averaging the scores over 6 days, rather than 7.

**Figure 2:**
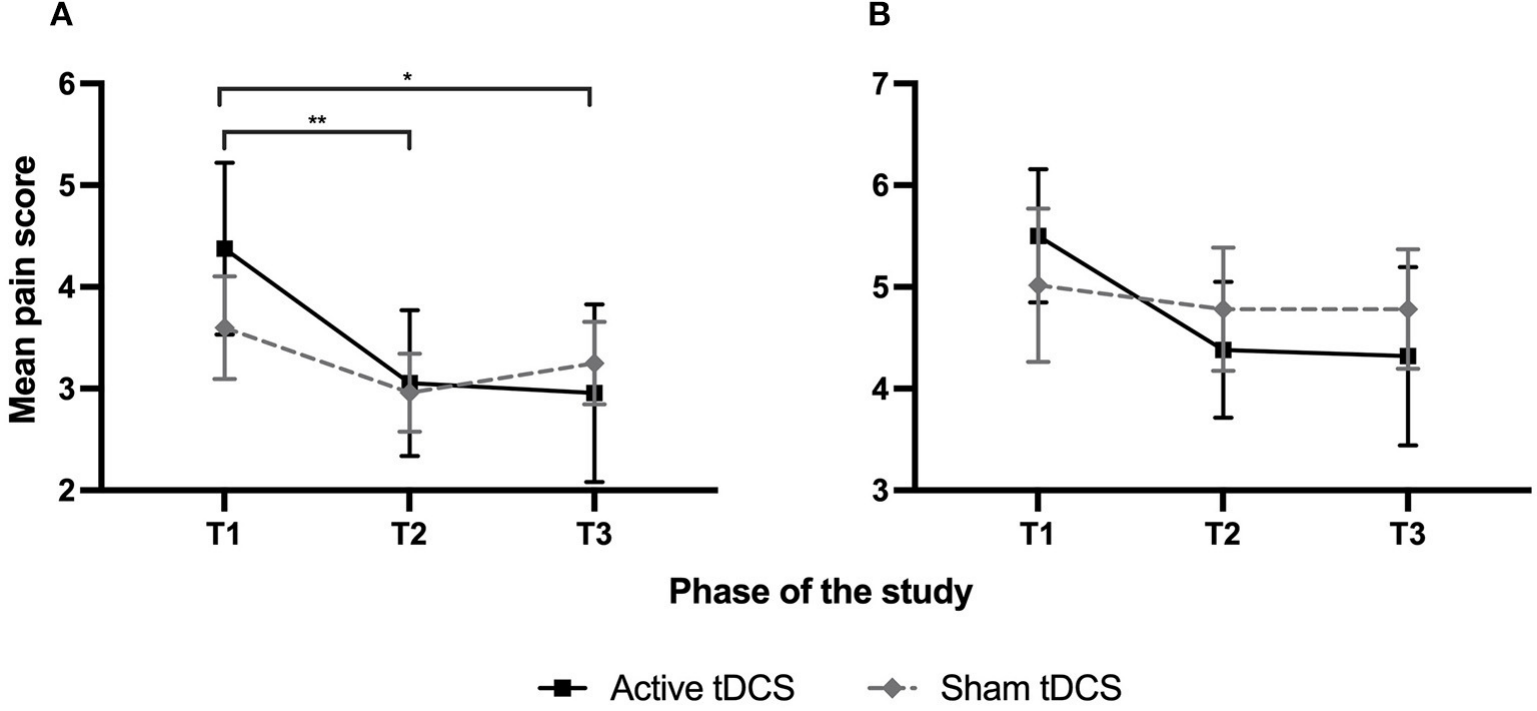
The average daily pain for sham and active treatment groups was gathered using the 0–10 NRS from the pain logbooks. T1 represents the 7 days of baseline, T2 corresponds to the 5 days of tDCS treatments, and T3 represents the 7 days of follow-up. Each point represents a group mean ± SEM (standard error of mean). **(A)** Daily average pain. **(B)** Daily maximal pain. *Statistically significant with *p* < 0.05; **Statistically significant with *p* < 0.005.

Pain intensity outcomes are presented in Figure 2. As it can be seen in Figure 2A, the daily average pain ratings decreased among the active tDCS group and remained unchanged among the sham tDCS group. This pattern of results was confirmed by Friedman tests, which revealed a significant effect of time in the active tDCS group (*p* = 0.006), but not in the sham tDCS group (*p* = 0.15). *Post-hoc* Wilcoxon signed-rank tests for the active tDCS group revealed that there was a significant reduction in the daily average pain during (T2; *p* = 0.003) and after (T3; *p* = 0.04) tDCS treatments, when compared to baseline (T1). For daily maximal pain scores (Figure 2B), they also tended to decrease in the active tDCS group, but results just failed to reach statistical significance (*p* = 0.05). No changes were observed for the sham tDCS group (*p* = 0.93).

**Figure 3:**
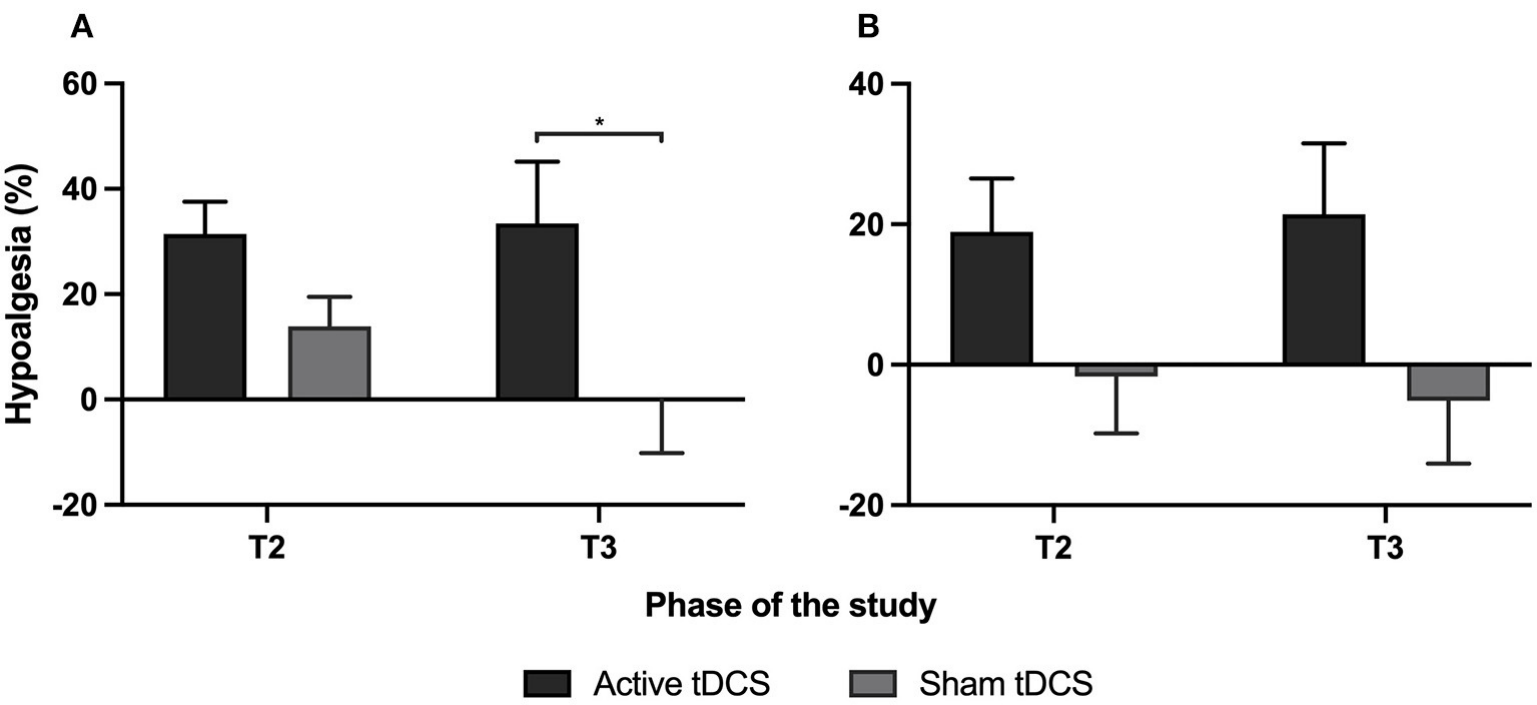
Percentages of hypoalgesia are calculated with **(A)** the daily average pain and **(B)** the daily maximal pain measured using the pain logbook. For each graph, the first two columns represent hypoalgesia during the week of tDCS treatments (comparing T2 to T1) and the second two columns represent hypoalgesia during the 7 days of follow-up (comparing T3 to T1). Each column represents mean ± SEM. *Indicates statistically significant (*p* < 0.05).

To better delineate the effect of active and sham tDCS, percentages of hypoalgesia were calculated and compared between the 2 groups (Figure 3). Active tDCS produced a reduction in daily average pain of 31% at T2 and 33% at T3. On the other hand, sham tDCS reduced daily average pain by 14% at T2 and slightly increased the pain by 1% at T3. *U* Mann–Whitney tests comparing the active tDCS and sham tDCS groups revealed a statistically significant difference between the two groups at T3 (*p* = 0.02), but not at T2 (*p* = 0.08). For daily maximal pain, active tDCS reduced the pain scores by 19% at T2 and 21% at T3, whereas sham tDCS slightly increased daily maximal pain scores by 2% at T2 and 5% at T3. *U* Mann–Whitney tests comparing daily maximal pain between both groups failed to reach statistical significance, both at T2 (*p* = 0.09) and T3 (*p* = 0.06).

### Pain-Related Outcomes

Results from the questionnaires evaluating pain quality (MPQ) and depicting all other pain-related outcomes are presented in Table 2. There were no missing data for these measures. Active tDCS generated a significant change for all outcomes (all *p* ≤ 0.02). *Post-hoc* Wilcoxon signed-rank tests revealed that active tDCS reduced MPQ, SF-BPI, BDI, BAI, PCS, and MPDSS scores at T2 compared to T1 (all *p* ≤ 0.04), suggesting that tDCS had positive effects on the qualitative aspects of pain, physical functioning, emotional functioning (depressive symptoms and anxiety), catastrophic thinking, and the body surface covered by pain. *Post-hoc* Wilcoxon signed-rank tests also revealed significant improvements at T3 when compared to T1 (all *p* ≤ 0.04), except for physical functioning (*p* = 0.31) and depressive symptoms (*p* = 0.09), suggesting that active tDCS produced a more persistent effect on the qualitative aspect of pain, anxiety symptoms, catastrophic thinking, and the body surface covered by pain. On the contrary, sham tDCS generated no change overtime for all pain-related outcomes (all *p* ≥ 0.08), except for the painful body surface that was significantly reduced at T2 and T3 when compared to T1 (both *p* ≤ 0.02). *U* Mann–Whitney tests revealed between-group differences for depressive symptoms and catastrophic thinking at T2 (both *p* ≤ 0.03).

**Table 2:** Pain-related questionnaires.

**Questionnaire**	**tDCS group**	**Scores**			***P value* ^†^**
		**T1**	**T2**	**T3**	
MPQ	Active	29.3 ± 12.0	13.6 ± 9.2	19.2 ± 14.4	0.00^**^
	Sham	27.8 ± 14.8	22.7 ± 13.2	21.5 ± 10.4	0.86
	*P value* ^‡^	0.67	0.10	0.67	
SF-BPI	Active	23.7 ± 12.2	10.8 ± 11.2	17.6 ± 16.0	0.01^**^
	Sham	23.3 ± 12.5	16.1 ± 11.3	16.5 ± 12.5	0.08
	*P value* ^‡^	0.89	0.24	0.88	
BDI	Active	4.2 ± 2.5	2.5 ± 2.1	3.2 ± 3.0	0.01^*^
	Sham	5.2 ± 3.4	5.3 ± 3.2	4.6 ± 3.1	0,15
	*P value* ^‡^	0.41	0.03^*^	0.29	
BAI	Active	12.2 ± 10.9	5.8 ± 7.0	6.4 ± 6.1	0.02^*^
	Sham	10.3 ± 7.8	8.8 ± 4.1	6.8 ± 4.2	0.17
	*P value* ^‡^	0.93	0.08	0.59	
PCS	Active	21.0 ± 10.0	6.8 ± 7.9	14.4 ± 13.6	0.00^**^
	Sham	23.3 ± 15.2	14.5 ± 9.7	13.2 ± 9.3	0.70
	*P value* ^‡^	0.84	0.01^*^	1.00	
MPDSS	Active	10.1 ± 5.4	5.6 ± 5.1	5.5 ± 5.6	0.02^*^
	Sham	11.4 ± 8.9	6.1 ± 7.2	6.0 ± 4.2	0.04^*^
	*P value* ^‡^	0.93	0.84	0.51	

*T1 = before the tDCS sessions, T2 = after the last tDCS session and T3 = 1 week after the end of the intervention*.

*Mean value ± standard deviation*.

† *Friedman tests were performed to detect intra-group differences*.

‡ *Mann-Whitney tests were performed to detect between-group differences*.

* *Statistically significant with P < 0.05*;

** *Statistically significant with P < 0.01*.

*tDCS, transcranial direct current stimulation; MPQ, McGill Pain Questionnaire; SF-BPI, Short Form of the Brief Pain Inventory; BDI, Beck Depression Inventory; BAI, Beck Anxiety Inventory; PCS, Pain Catastrophizing Scale; MPDSS, Margolis Pain Drawing and Scoring System*.

## Discussion

The present study aimed to evaluate the efficacy of tDCS on pain in older individuals living with chronic musculoskeletal pain. Overall, the results of this randomized, double-blind, sham-controlled trial indicate that tDCS is an effective approach for reducing chronic musculoskeletal pain and improving pain-related outcomes in elderly individuals. It is important to reiterate that the present study includes and builds on data coming from a previous pilot study (35); the first part of the trial and the outcome data generated hence contribute to the final analyses (71). Eldridge et al. describe pilot studies as a subset of feasibility studies (72). In these types of studies, not only are feasibility issues assessed, but their results also allow researchers to optimize the methods used in the main study to avoid wasting valuable research resources and recruiting participants into a trial that may not succeed (71–73). In the specific case of our pilot study, the methods and objectives were modified to remove all the elements related to sleep measurement, given the negative results observed for this outcome (35).

We observed a significant reduction in the daily average pain ratings among the active tDCS group during treatment (T2) and in the following week (T3) when compared to baseline (T1). Importantly, the percentages of hypoalgesia measured at T2 and T3 were found to be clinically significant, suggesting that patients with chronic pain may actually benefit from pain reduction achieved with tDCS (51, 70). Our observations are consistent with the results of previous work looking into the efficacy of tDCS in older adults suffering from chronic pain, all of which obtained positive results (29, 30, 74–78). Although all these studies point in the same direction, the magnitude of pain reduction and the long-lasting effect vary from one study to another. In our case, daily average pain intensity scores decreased by 1.32 points and 1.41 points on the 0–10 NRS during the week of treatment and in the week after the last tDCS session, respectively. These results are similar to those of Ahn et al. (knee OA pain) (74) who reported reductions of 18.5/100 after tDCS sessions and of 16.4/100 at the 1-week follow-up, but are inferior to those obtained by Concerto et al. (plantar fasciitis) (30) who observed a reduction of approximately 2.6/10 during the week of tDCS sessions and 2.85/10 on the week after the end of tDCS sessions. Most of the tDCS studies conducted so far in the elderly focused on knee OA. Although OA is a prevalent condition in the elderly, it will be important to look at the effects of tDCS on other painful conditions in the future (28, 29, 74–78).

Our results also showed that active tDCS improved all pain-related measures during the week of treatment (T2) when compared to baseline (T1). Physical functioning and pain quality are two of the most studied pain-related outcomes in trials looking into the effect of tDCS in older adults (29, 30, 74, 78). In our study, physical functioning, as measured with the BPI, showed an improvement of 12.9 points at T2. These results differ from those obtained by Tavares et al. (78)—the only other study to evaluate physical functioning with the BPI—who reported a decrease of only 2.27 points after 15 sessions of tDCS. The observations of Tavares et al. are consistent with those of Ahn et al. (74)—who used the Western Ontario and McMaster Universities Index (WOMAC) to evaluate the impact of pain on physical functioning and who failed to observe significant improvement following five sessions of tDCS—but contrasts with the results of four other studies, all of which observed significant improvements in WOMAC scores following tDCS treatments (28, 29, 76, 77).

In the present study, the improvements observed in all pain-related outcomes persisted over time and remained statistically significant 1 week after the last tDCS intervention (T3) when compared to baseline (T1), except for physical functioning and depressive symptoms. This follow-up period, although relatively short, is one of the strengths of this study, as most of the other studies looking into the effect of tDCS in older adults did not plan any follow-up assessments (28, 29, 75–77). From the only 3 studies which included a follow-up period, 1 reported no lasting effect of tDCS (78) and 2 reported a sustained reduction of pain intensity (30, 74). One of these studies also observed an improvement in physical functioning and anxiety symptoms during a 1-week follow-up (30).

All participants included in the analyses completed all 5 tDCS sessions. Only 2 participants dropped out from the study: one before starting the first tDCS intervention (death of a loved one) and the other after attending 3 tDCS sessions (withdrawal of driver's license). Like most studies of non-invasive brain stimulation in depression or chronic pain [> 90%, according to Thibaut et al. (79)], no strategy was initially planned for participants who missed the tDCS sessions (79). Thibaut et al. suggested that a maximum of 20% of missing sessions should be allowed before excluding a participant and that these sessions should be replaced at the end of the stimulation period (79). Following these recommendations, the two participants who dropped out (who had missed 40% and 100% of their tDCS sessions), were excluded from the analyses. Taking into consideration the reasons for dropping out, we decided not to conduct intention-to-treat analyses, so as not to unduly underestimate the effect of the intervention.

Transcranial direct current stimulation was well tolerated in our participants (no important or severe side effects). Of all the tDCS sessions, only 20% were associated with benign side-effect (92% classified as mild and 8% as moderate). These observations correspond to the aggregation of tDCS experiences in humans, presented by Bikson et al., which revealed that tDCS did not produce any serious adverse effect or irreversible injury across over 33,200 sessions on 1,000 participants (36). Tingling was the most common effect observed in our participants, followed by, but far ahead of, headache and sensations of heat under the electrodes, which were both reported twice. Tingling was also reported by Concerto et al. (30), Ahn et al. (74), and Tavares et al. (78). Interestingly, Tavares et al. noted that tingling sensation and headache were significantly higher among their sham tDCS group (78).

Blinding was successful as only 13 out of 24 participants were able to correctly guess the type of stimulation they received, a ratio which is no better than chance or coincidence. Our results also suggest that our evaluator, who correctly guessed 14 times out of 24 in the group to which the participants belonged, was successfully blinded. Other tDCS studies conducted in the elderly also reported successful blinding of the participants, but did not discuss evaluators' blinding (28, 77, 78). O'Connell et al. (80) suggested that the use of a sham tDCS treatment applied at an intensity of 2 mA is hardly attainable, given that evaluators are often able to observe skin redness under the reference electrode following active tDCS (80). In the present study, all participants (including those who received sham tDCS) presented some redness under the reference electrode, an element that probably contributed to the successful blinding of the evaluator. Although blinding of participants and evaluators is not perfect, a publication of Brunoni et al. (81) revealed that there are no important blinding-related biases in tDCS clinical trials when using parallel designs, partly because this type of study design (vs. crossover design) does not allow the participants to compare the procedures and sensations felt during active and sham conditions.

This study had some limitations. First, participants were met by the evaluator on the last day of tDCS treatment to fill the questionnaires. Although the evaluator was not able to distinguish between the active and sham stimulation, some authors report that skin redness can help guess the type of stimulation up to 30 min after the end of stimulation (65, 78, 80, 82). According to these authors' observations, Brunoni et al. (66) suggested that breaking blinding should be avoided by having a backup blinded evaluator available to substitute an evaluator who notices evidence of redness skin of a patient. In the present study, skin redness was similar between the two groups (active and sham). A second limitation is that the short follow-up period does not provide data to draw conclusions regarding the long-term efficacy of tDCS. Some studies conducted on younger populations showed that 5 sessions of tDCS can produce a significant hypoalgesic effect that can last up to 6 months after the end of stimulations (83–85). In older adults, the longest follow-up period for 5 tDCS sessions demonstrated that knee OA pain can be reduced up to 3 weeks after the last tDCS intervention (74). Third, our participants were asked to keep their medication stable for the duration of the study, and no participant was excluded based on their medication consumption. However, observations from a review suggest that multiple classes of medications may impact the effect of tDCS, and that tDCS trials should carefully consider what types of medications are allowed for their participants (86). Nevertheless, there are ethical considerations when conducting a study with the elderly suffering from chronic pain, and our research team was not comfortable asking participants to stop medication consumption. There are also benefits to not exclude these participants (e.g., increased external validity). Fourth, there was no statistical correction applied for the *post hoc* tests. Given the relatively small number of participants and the fact that sample size calculation was done without considering such analyses, we refrained from applying corrections for the *post hoc* tests to reduce the risk of Type II errors. Finally, although many changes in pain intensity and pain-related outcomes are statistically significant, these results should be interpreted with caution as their clinical significance varies according to the thresholds set by different studies (69, 70).

### Conclusion

The present study provides additional evidence on the efficacy of tDCS for reducing chronic musculoskeletal pain in the elderly. Our intervention (5 sessions of tDCS) reduced pain intensity and quality and improved many pain-related outcomes, such as physical functioning, emotional functioning (symptoms of anxiety and depression), catastrophic thinking, and body surface covered by pain. At this stage, and after the accumulation of evidence from all these small sample studies confirming the beneficial effect of tDCS in elders suffering from chronic musculoskeletal pain, we believe that the field is ready for the implementation of a large pragmatic multicenter study to confirm these promising results and better define the role tDCS could have in the prise en charge of this population.

## Data Availability Statement

The data that support the findings of this study are available from the corresponding author upon request.

## Ethics Statement

This study was reviewed and approved by the Comité d'éthique de la recherche du CIUSSS de l'Estrie-CHUS. The patients/participants provided their written informed consent to participate in this study.

## Author Contributions

MPH wrote the research protocol and the present manuscript, collected most of the data, and realized statistical analysis. MM and FH helped in data collection and revised this manuscript. ID helped with recruitment and data collection and revised the manuscript. ER helped to develop the research project and revised the manuscript. GL developed the research project, supervised the trial and the data analysis, and revised the manuscript. All authors contributed to the article and approved the submitted version.

## Funding

GL is supported by the Fonds de Recherche en Santé (FRQ-S, Québec, Canada). The project was partially supported by the Center d'excellence en neurosciences de l'Université de Sherbrooke (CeNUS, Québec, Canada) and an internal start-up fund from the Research Center on Aging (Initiatives stratégiques du Center de recherche sur le vieillissement, Québec, Canada).

## Conflict of Interest

The authors declare that the research was conducted in the absence of any commercial or financial relationships that could be construed as a potential conflict of interest.

## Publisher's Note

All claims expressed in this article are solely those of the authors and do not necessarily represent those of their affiliated organizations, or those of the publisher, the editors and the reviewers. Any product that may be evaluated in this article, or claim that may be made by its manufacturer, is not guaranteed or endorsed by the publisher.
